# Evolving models for medical physics education and training: a global perspective

**DOI:** 10.2349/biij.4.1.e16

**Published:** 2008-01-01

**Authors:** P Sprawls

**Affiliations:** Sprawls Educational Foundation, Montreat, North Carolina, United States

**Keywords:** Effective education, efficient learning activities, technology enhanced education, shared resources

## Abstract

There is a significant need for high-quality medical physics education and training in all countries to support effective and safe use of modern medical technology for both diagnostic and treatment purposes. This is, and will continue to be, achieved using appropriate technology to increase both the effectiveness and efficiency of educational activities everywhere in the world. While the applications of technology to education and training are relatively new, the successful applications are based on theories and principles of the learning process developed by two pioneers in the field, Robert Gagne and Edgar Dale.

The work of Gagne defines the different levels of learning that can occur and is used to show the types and levels of learning that are required for the application of physics and engineering principles to achieve appropriate diagnostic and therapeutic results from modern technology. The learning outcomes are determined by the effectiveness of the learning activity or experience. The extensive work of Dale as formulated in his Cone of Experience relates the effectiveness to the efficiency of educational activities. A major challenge in education is the development and conduction of learning activities (classroom discussions, laboratory and applied experiences, individual study, etc) that provide an optimum balance between effectiveness and efficiency. New and evolving models of the educational process use technology as the infrastructure to support education that is both more effective and efficient.

The goal is to use technology to enhance human performance for both learners (students) and learning facilitators (teachers). A major contribution to global education is the trend in the development of shared educational resources. Two models of programs to support this effort with open and free shared resources are Physical Principles of Medical Imaging Online (http://www.sprawls.org/resources) and AAPM Continuing Education Courses (http://www.aapm.org/international).

## INTRODUCTION

Medical practice and healthcare facilities in most countries are becoming increasingly effective in diagnosing and treating many diseases, thanks to advances in science and technology for both diagnostic and therapeutic applications. In order for these advances to benefit citizens anywhere in the world, the medical technology has to be available and accessible, and highly-educated and trained medical professionals have to be able to utilise the technology for maximum effectiveness.

Medical physicists have the knowledge to ensure optimum and safe utilisation of modern medical equipment for the benefit of all patients. This is achieved through applied clinical activities such as treatment planning and imaging procedure optimisation, education and training of other medical professionals such as physicians and technologists, evaluation of equipment performance, risk analysis, and management of quality and safety activities.

There are two specific dynamics that have an impact on the effectiveness of education and training activities in a specific geographic region. One is the many rapid advances in the science and technology that require almost constant updating of knowledge, experience, and educational materials on a local basis. The other is the need to transfer knowledge from the few centres of experience with new technologies and methods to the many worldwide locations for clinical application. These needs will only be met by transiting to new models of the education and training process where state-of-the-art technology is used to enhance human performance of both learners (students) and learning facilitators (teachers). The goal is to produce enriched learning environments on a global basis to support highly effective learning activities.

We will now review the characteristics of learning environments, especially with respect to their *effectiveness* and *efficiency*, as described by two of the major pioneers in the educational process and then analyse two models that are making major contributions on a global basis.

## EFFECTIVE LEARNING

In order to contribute to improved healthcare, the knowledge of medical physics must be applied in the clinical environment to plan and optimise procedures, analyse performance, solve problems, and other creative activities. This requires a higher level of learning than might be required for adequate performance on many written examinations.

**Figure 1:**
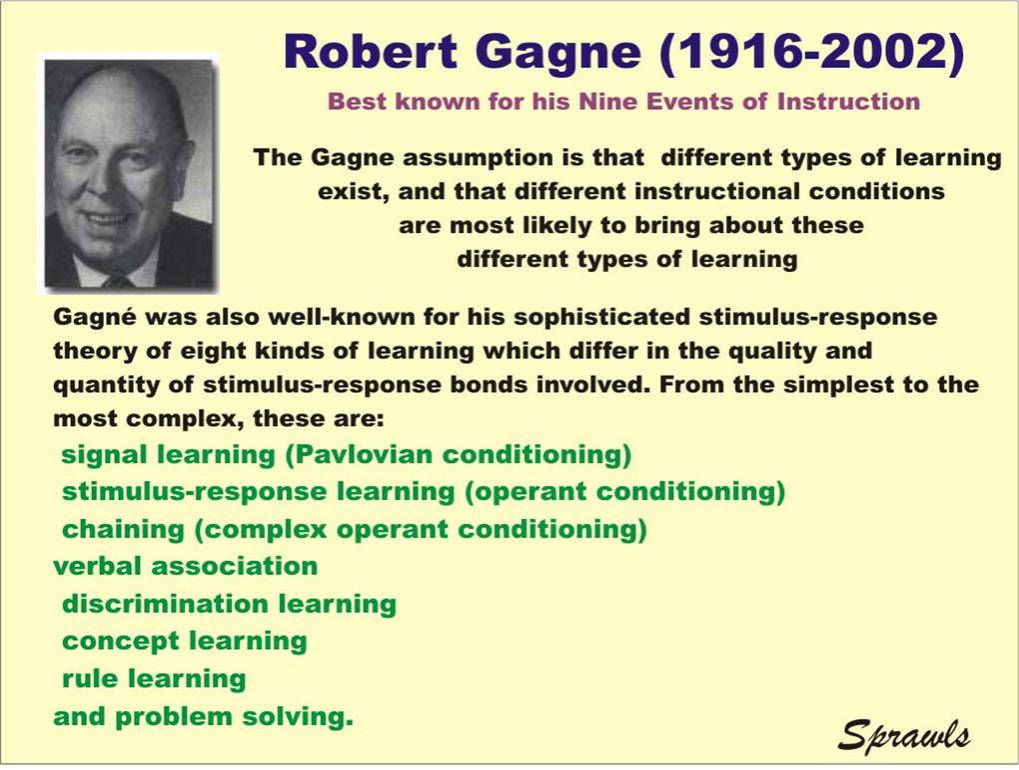
Robert Gagne.

Robert Gagne, introduced in Figure 1, provides an analysis of the learning process which defines the different types of learning.

### Gagne's Principles Applied to Medical Physics Education

**Figure 2:**
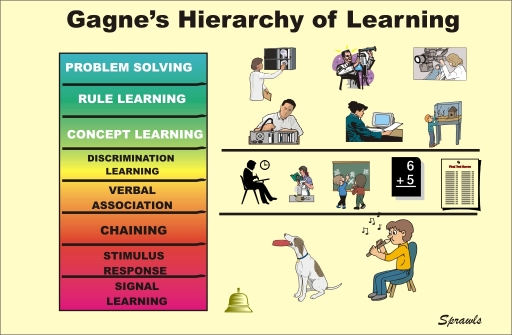
Gagne's Levels of Learning as Illustrated by the Author.

A major contribution of Gagne that applies to medical physics education is the formulation of the hierarchy and levels of learning as illustrated in Figure 2.

Not all learning activities result in achieving the same abilities to perform specific functions. The application of medical physics in the clinical environment generally requires the higher levels of learning as illustrated above.

The level of learning achieved and the ability to perform specific functions depends on the *effectiveness* of the learning activity. While it is desirable for a learning activity, such as a classroom discussion, to be highly effective there is a major compromise that must be considered. That is the *efficiency* of the learning activity in terms of required resources such as personnel time and effort, institutional facilities, and financial costs as illustrated below.

## RELATIONSHIP OF EFFECTIVENESS AND EFFICIENCY OF LEARNING ACTIVITIES

**Figure 3:**
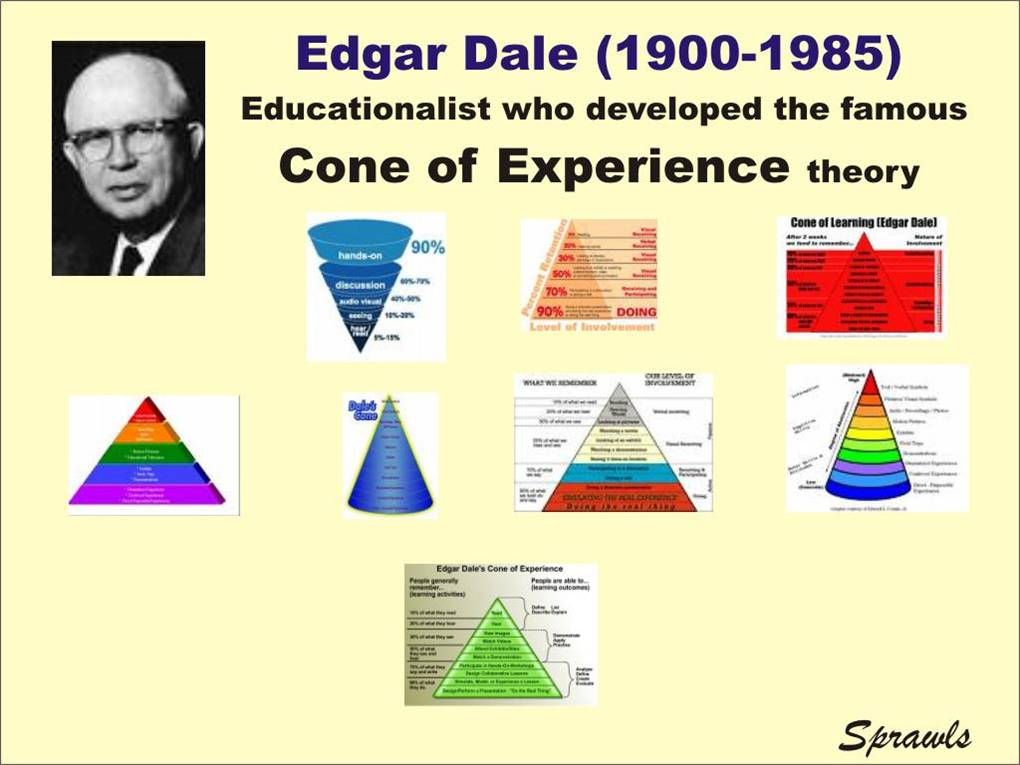
Edgar Dale's Cone of Experience has been interpreted and published in many different forms as illustrated here.

This significant relationship between effectiveness and efficiency of learning activities was formulated by Edgar Dale and described with the Cone of Experience which has been published in different forms as illustrated in Figure 3.

### Dale's Cone of Experience

**Figure 4:**
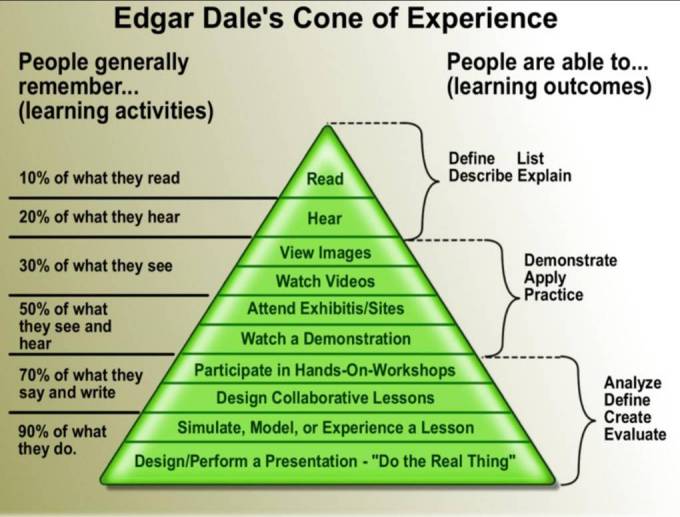
Dale's Cone of Experience Illustrating Different Types of Learning.

Dale's cone of experience is shown in more detail in Figure 4.

As one can see, the cone of experience organises learning experiences and activities in terms of their effectiveness in producing learning outcomes.

The learning outcomes that are crucial to clinical medical physics (analyse, create, evaluate, problem-solve, etc.) are best developed by experiences and learning outcomes at the base of the cone.

### The Cone of Experience Applied to Medical Physics Education

**Figure 5:**
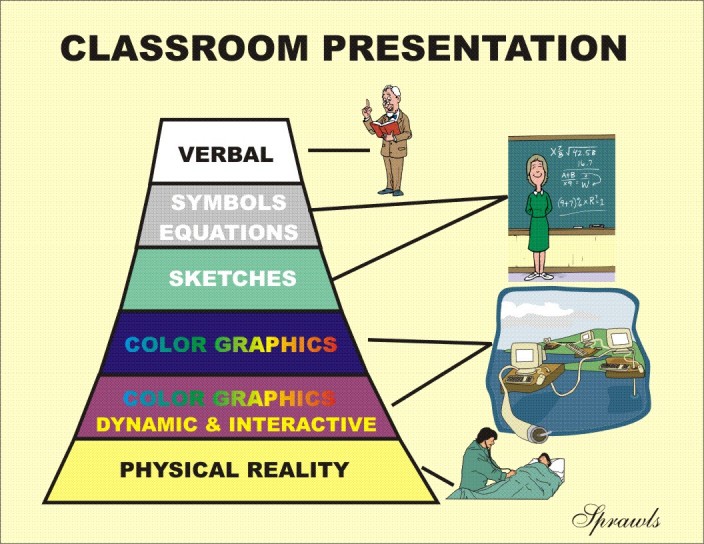
Medical physics learning activities range from listening to verbal lectures at the top of the cone to direct contact, interaction, and experience with the physical items and conditions that form the base.

The concept of the cone of experience applied more specifically to medical physics education is shown in Figure 5.

### Relationship of the Cone of Experience to Effectiveness and Efficiency

**Figure 6:**
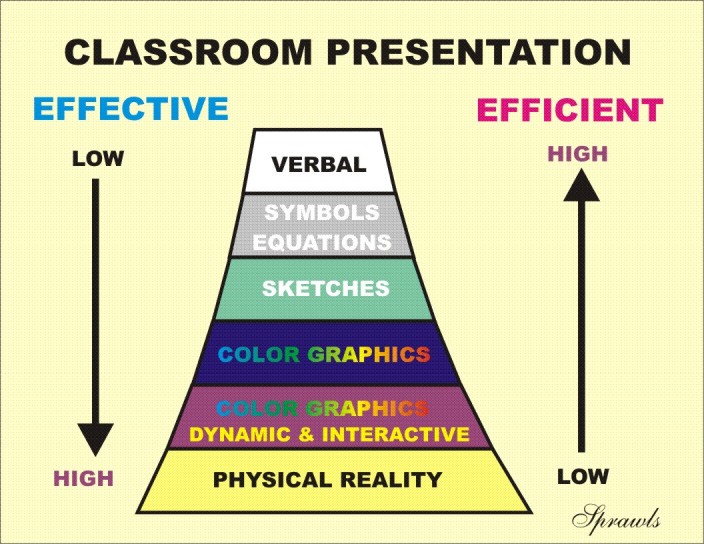
The real significance of the cone of learning activities, especially for applied medical physics, is that effectiveness of the learning experience to produce the higher levels of learning and necessary outcomes are generally the least efficient and most costly to produce.

A major question is why a specific type of learning activity is selected and used. The answer is found when the characteristic of efficiency is added to the cone as shown in Figure 6.

## EFFICIENCY OF EDUCATIONAL ACTIVITIES

**Figure 7:**
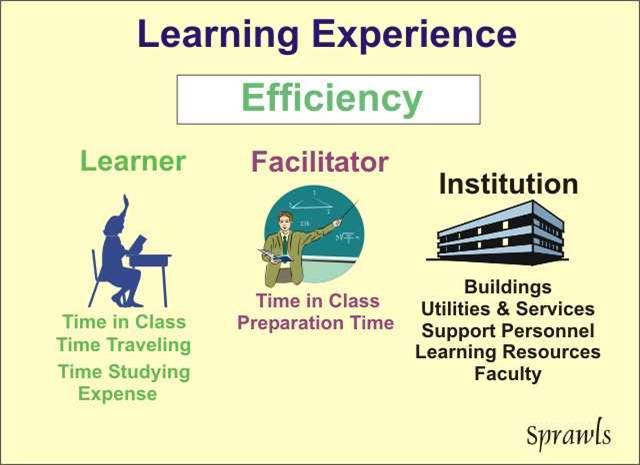
Factors that determine the efficiency of educational activities.

Many factors that have an effect on efficiency are illustrated in Figure 7.

Any learning activity requires resources and it is this overall requirement that determines the efficiency of the activity. As has been observed above, learning activities that can be highly effective with outcomes to support applied clinical medical physics (the lower section of the *Cone of Experience*) require significant resources, and are therefore not the most efficient when compared to some other educational methods.

### The Role of Digital Technology

**Figure 8:**
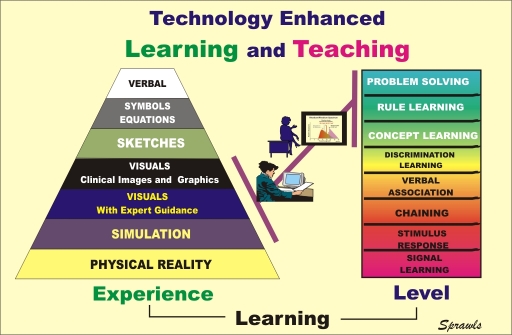
Technology, especially through the global availability of high-quality visuals and images is contributing to more effective learning.

The long-standing challenge between the *efficiency* and *effectiveness* of education activities is now being reduced through the availability of state-of-the-art digital technology. One of its contributions that relates to the cone of experience and the levels of learning is illustrated in Figure 8.

Technology is now making a major contribution to more effective medical physics education by providing high-quality visual representations to enrich the learning environments and move the learners closer to the physical reality that they are studying.

High-quality visuals have the capability of ‘making the invisible now visible’ (radiation, atomic structures, etc.), showing relationships and interactions, and illustrating virtually all aspects of medical imaging.

The increased efficiency comes from the sharing of the digitised resources so that local learning facilitators (teachers) and learners (students) can devote their time and effort to a more productive learning process.

### The Traditional Classroom Learning Environment Model

**Figure 9:**
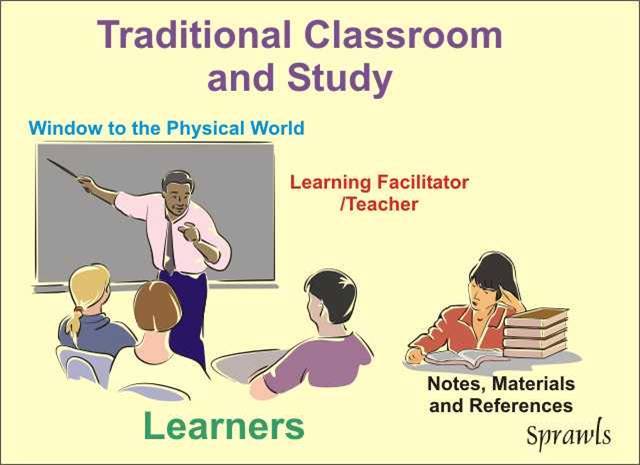
The traditional classroom has been the best available learning environment from the standpoint of efficiency. Large groups of learners can be brought together and "taught" by the highly efficient (but not so effective) lecture process.

For centuries, the usual learning environment has been the classroom as illustrated in Figure 9.

**Figure 10:**
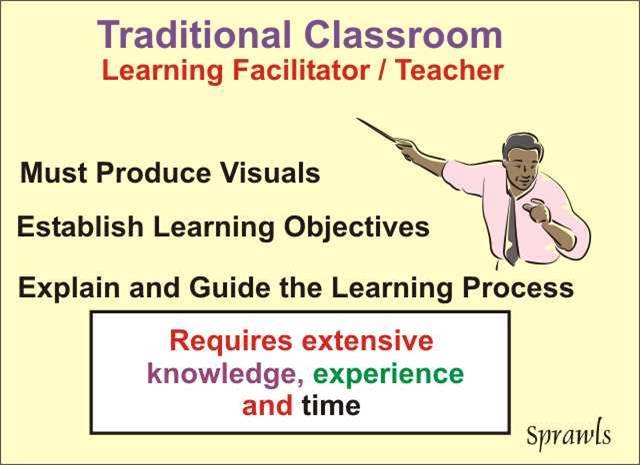
For the learning facilitator/teacher there is the need to produce visuals (windows to the physical world) and to have an up-to-date knowledge of applied medical physics.

While the traditional classroom process continues to be useful for many topics, especially those that are conveyed through audio media (music, languages, etc.), it presents a major challenge for effective medical physics education for both the learners and the learning facilitators as illustrated in Figure 10.

The production of high-quality visuals that can connect the learner to the physical world (medical physics) about which they are learning requires extensive time, talent, knowledge, experience, and technical resources that are not generally available to the traditional classroom teacher.

Because of the rapid advances in medical physics and the associated technology for both imaging and therapy, and the migration of these around the world, the local learning facilitator is challenged with having to keep ‘up-to-date’ through continuing education and lifelong learning.

### The Learner in the Traditional Classroom Model

**Figure 11:**
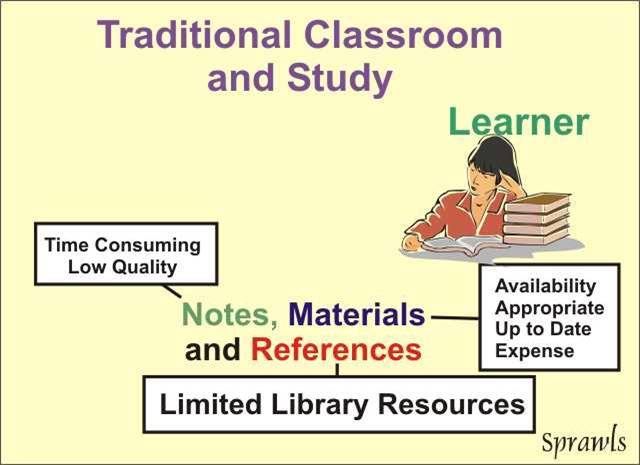
Factors that limit both the efficiency and effectiveness of classroom learning and individual study before the availability of technology.

The learner in the traditional classroom model is first challenged with the necessity of recording, in written form, both the visuals and spoken words and then using the materials later for effective learning as illustrated in Figure 11.

### Technology Enhanced Education

Many of the challenges and short-comings of traditional education methods, especially in medical physics, are being reduced by innovative applications of technology.

The desired role of technology is not to replace the learning facilitator/teacher but to enhance human performance, through increased effectiveness and efficiency, of both the learner/student and the learning facilitator/teacher*.*

**Figure 12:**
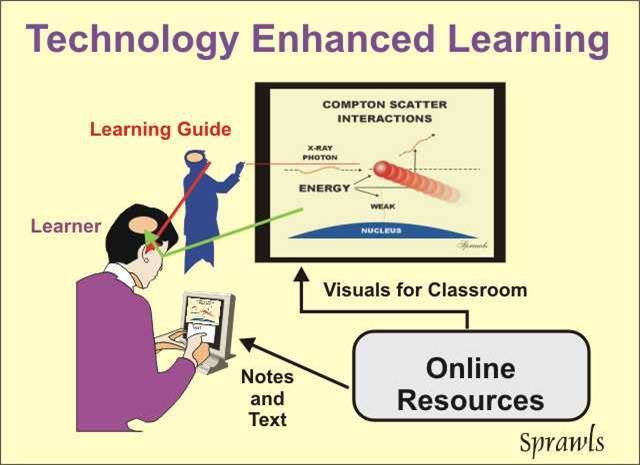
The foundation of most technology enhanced learning is the availability of a comprehensive collection of digital resources to support the different activities with the goal of making them both more effective and efficient. This is best achieved by shared resources made available on a global basis by distribution over the Internet or through the physical distribution of recorded digital media like CD-ROMs.

This is, and will continue to be, achieved through evolving models of the educational process that combine the advantages and values of both technology and humans. Figure 12 shows how this is achieved in a variety of medical physics academic courses and for continuing education.

When high-quality visuals are available from an online resource, the local learning facilitator can devote time to guiding and leading the learning process with their personal knowledge and experience. This can be a highly effective learning activity producing the desired outcomes for clinical medical physics applications because it combines high-quality visuals to enrich the learning environment with the experience of the local learning facilitator. It is also very efficient for the learning facilitators because their effort can be directed to engaging the learner and guiding the learning process rather than having to produce visuals and other related materials.

The learner is now "seeing" much of the physical universe rather than hearing it described in words, which results in a much more effective learning experience and higher levels of learning. When the visuals and related descriptive materials are also available to the learner for later study and review, the total learning activity becomes much more efficient.

## ENRICHING THE LEARNING ENVIRONMENT WITH SHARED RESOURCES

One of the greatest needs in medical physics education and training is to enrich the local learning environments everywhere in the world with the up-to-date experience associated with the developments in science and technology. A model of how this can be achieved is illustrated below.

**Figure 13:**
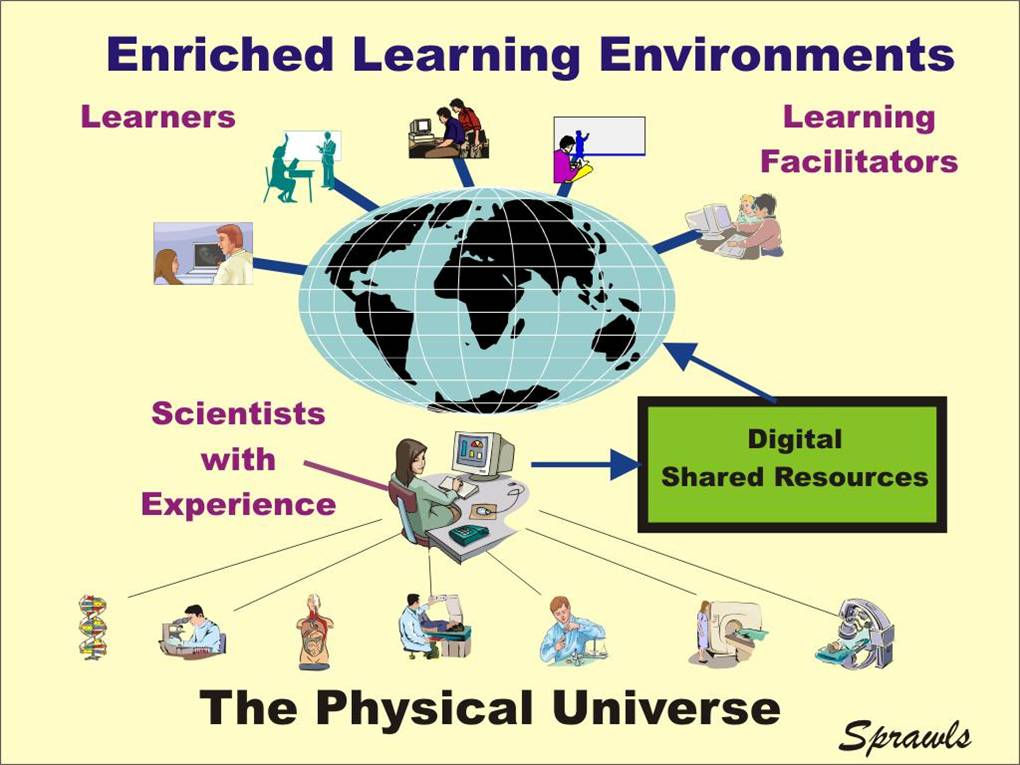
Shared resources developed in this process enrich the learning environments for both courses provided by academic institutions and by organisations for continuing education and lifetime learning.

The requirement is that physicists, other scientists, and medical professionals who have the experience with the various technologies and applications transfer their knowledge into digital resources that are shared with both learners and learning facilitators on a global basis as illustrated in Figure 13.

We will now consider a model for each of these applications.

### The Physical Principles of Medical Imaging Online

**Figure 14:**
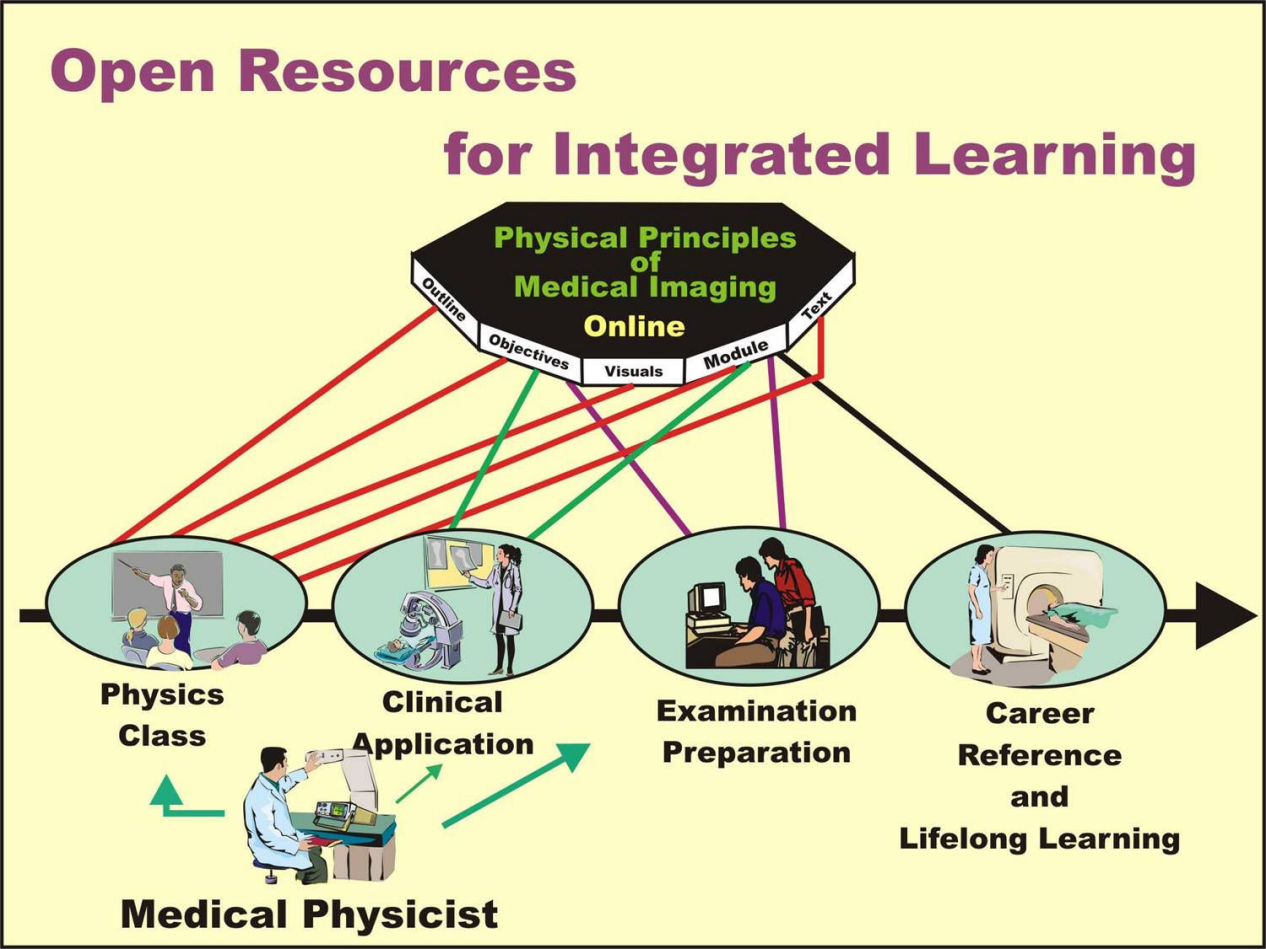
The types of resources and the learning activities supported by the Physical Principles of Medical Imaging Online at http://www.sprawls.org/resources.

The *Physical Principles of Medical Imaging Online* (PPMI) is a multifaceted shared resource as illustrated in Figure 14.

The purpose of PPMI Online is to support and enrich each step in the integrated learning process of the physics of medical imaging for medical physicists, radiologists, and other medical imaging professionals. In most applications it is integrated into, and used as a resource for, courses provided by institutions under the direction of the local medical physics faculty.

### Open Resources for Medical Physics Continuing Education

**Figure 15:**
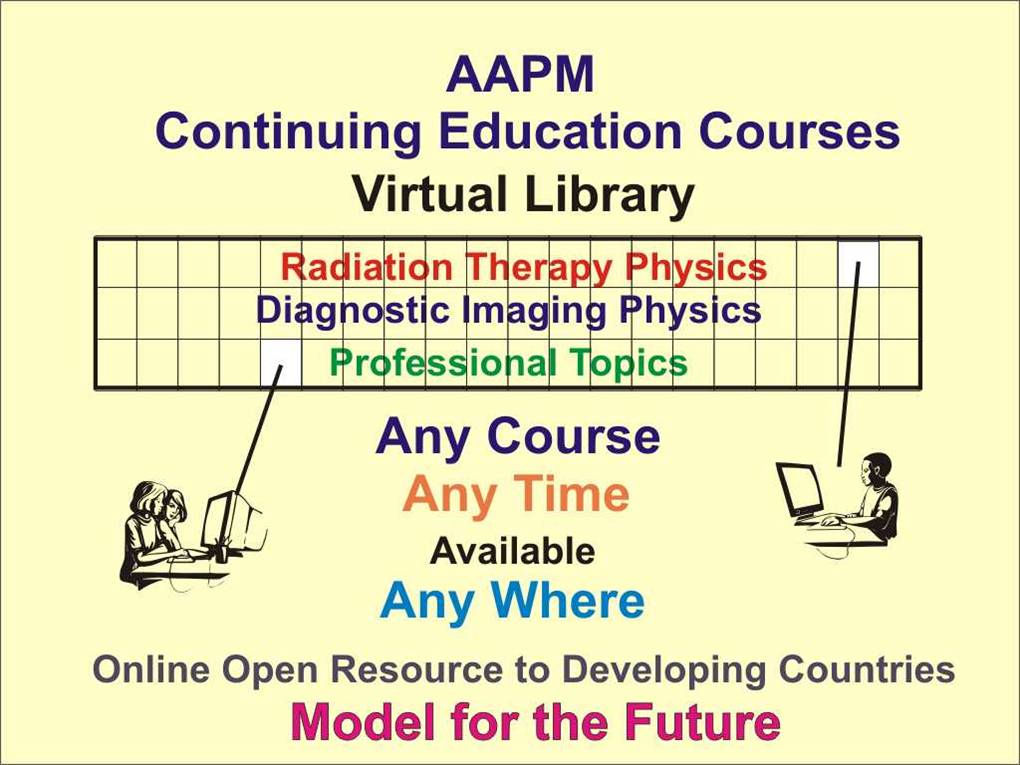
The AAPM Virtual Library provides continuing medical physics education that is available anywhere in the world through http://www.aapm.org/international.

The American Association of Medical Physicists (AAPM) provides many continuing education activities each year. These include approximately 50 courses presented during each AAPM Annual Meeting and also the Summer School devoted to a specific topic of interest each year. These courses and the proceedings of the Summer School are now available through the online Virtual Library as illustrated in Figure 15.

In addition to being available to AAPM members, the courses are now available at no cost to all medical physicists in developing countries who register to be an AAPM Developing Country Educational Associate (DCEA) through the international portal web site (http://www.aapm.org/international/).

### SUMMARY AND CONCLUSIONS

There is a significant need for high-quality medical physics education and training in all countries to support effective and safe use of modern medical technology for both diagnostic and treatment purposes.

This is, and will continue to be, achieved using appropriate technology to increase both the effectiveness and efficiency of educational activities everywhere in the world. While the applications of technology to education and training are relatively new, the successful applications are based on theories and principles of the learning process developed by two pioneers in the field, Robert Gagne and Edgar Dale.

The appropriate goal is to use technology to enhance human performance for both learners (students) and learning facilitators (teachers).

Two models of programs to support this effort with open and free shared resources are:

- Physical Principles of Medical Imaging Online at: http://www.sprawls.org/resources
- AAPM Continuing Education Courses available through: http://www.aapm.org/international

